# Identification of Acetomycin as an Antifungal Agent Produced by Termite Gut-Associated Streptomycetes against *Pyrrhoderma noxium*

**DOI:** 10.3390/antibiotics13010045

**Published:** 2024-01-03

**Authors:** Cherrihan Adra, Trong D. Tran, Keith Foster, Russell Tomlin, D. İpek Kurtböke

**Affiliations:** 1 School of Science, Technology and Engineering, University of the Sunshine Coast, Maroochydore BC, QLD 4558, Australia; cherrihan.adra@research.usc.edu.au (C.A.); ttran1@usc.edu.au (T.D.T.); 2 Centre for Bioinnovation, University of the Sunshine Coast, Maroochydore BC, QLD 4558, Australia; 3 Brisbane City Council, Program, Planning and Integration, Brisbane Square, Level 10, 266 George Street, Brisbane, QLD 4000, Australia; kfosteradsl@bigpond.com (K.F.); russell.tomlin@brisbane.qld.gov.au (R.T.)

**Keywords:** acetomycin, actinomycetes, *Streptomyces*, streptomycetes, *Pyrrhoderma noxium*, termite gut symbiosis, antifungal compounds, metabolomics, nuclear magnetic resonance

## Abstract

Plant fungal pathogen *Pyrrhoderma noxium* is responsible for the destructive and invasive disease of brown root rot currently affecting the city of Brisbane, Australia. In order to address this issue, environmentally friendly and safe alternatives to chemical control are preferred due to the city’s public setting. Antifungal natural products are ideal candidates as biological control alternatives and can be detected through investigating the metabolomes of microbial symbionts. Within this study, an NMR-based metabolomics approach was applied to fermentation extracts obtained from 15 termite gut-associated streptomycetes. By analysing the NMR spectra, six of the extracts which displayed similar chemical profiles exhibited antifungal activity against the *P. noxium* pathogen. The major compound within these extracts was identified as acetomycin using NMR and X-ray crystallography analyses. This is the first reporting of acetomycin as a potential natural product fungicide, particularly as an antifungal agent against *P. noxium*. Inhibitory activity was also found against other important fungal crop pathogens, including *Aspergillus niger*, *Botrytis cinerea*, and *Alteranaria alternata*. Further experimentation using a woodblock test found inhibitory activity on the growth of the *P. noxium* pathogen for up to 3 weeks and a significant difference in the integrity of the woodblocks when conducting compression strength tests after 6 weeks. Therefore, acetomycin may be used as a biological control agent and natural product fungicide against *P. noxium*.

## 1. Introduction

Plant pathogens play a major role in the destruction of natural resources within agriculture, leading to a reduction in annual food level production of up to 40% worldwide [1,2]. One pathogen known to cause such issues for plantations and orchards is *Pyrrhoderma noxium* (*P. noxium*). *Pyrrhoderma noxium*, formally known as *Phellinus noxius* [3], is a widespread tropical fungal phytopathogen that causes brown root rot in a large variety of tree species (over 250 species) [4]. It predominately infects the roots and lower trunk of the tree, resulting in crown dieback, chlorosis, discolouration, establishment of a “brown stocking”, and, ultimately, tree death [5,6,7]. Due to the decay within the roots, the trees easily fall down, which can be a threat to public safety [8]. Currently, the city of Brisbane in Queensland, Australia, is facing the spread of this pathogen and the subsequent destruction of many of the amenity trees [9,10]. Numerous privately owned and park trees are being killed, including the heritage Moreton Bay fig trees, poinciana, jacarandas, and hoop pines [9,11]. As chemical control methods are deemed no longer suitable, particularly within public locations due to their harmful effects and persistent nature, alternative methods are required [12]. A substantial amount of research has regarded the use of biological control agents as a promising alternative method for controlling such fungal disease in sustainable agricultural practices and within public settings [13].

Actinomycetes are Gram-positive bacteria and important biotechnological resources due to their ability to produce bioactive secondary metabolites, including antifungal agents [14]. In fact, 70% of antimicrobial substances are produced from the members of the genus *Streptomyces* from the actinomycetes [15]. It has been found that streptomycetes as biological agents are effective alternatives for disease management strategies, have reduced environmental impacts, and are compatible with organic agriculture [16]. A review study conducted by LeBlance (2022) compiled data from 160 studies that assessed the effectivity of streptomycetes biological control agents against fungal plant pathogens of agronomic and horticultural crops and found that they significantly reduced disease prevalence by 56% [16]. The major efficacy behind this genus in plant protection is the production of bioactive secondary metabolites such as antifungal agents, biopesticides, and bioinsecticides [17]. Streptomycetes have high guanine (G) + cytosine (C) content and a complex lifestyle that involves morphological differentiation. Such physiological change is believed to prompt the production of various secondary metabolites as an adaptive way to deal with stress [18]. Recent interest has been shown towards unique or extreme environments for the isolation of specialised streptomycetes with the ability to produce novel structures with potent biological activities [19]. One such resource is from insect guts that contain communities of transient and symbiotic microorganisms [20]. Termites feed mostly on wood and litter tissue, meaning they would frequently encounter various fungal pathogens and would, therefore, require defense mechanisms to protect themselves against invading pathogens [9,21]. It has also been reported that actinomycetes are among the most dominant taxonomic group within the guts of termites, with a majority of the actinomycetes belonging to the genus *Streptomyces* [22]. Therefore, termite gut-associated streptomycetes may produce new or novel secondary metabolites with the ability to control phytopathogenic fungi such as *P. noxium*.

Metabolomics is a powerful tool to describe the comprehensive metabolic status of a microorganism through analysis of the diversity and abundance of its small-molecule metabolites [23]. This method is becoming increasingly popular as it provides a systematic profiling of metabolites with potential in drug development, molecular medicine, and other biotechnological fields [24]. One technique to achieve this is through Nuclear Magnetic Resonance (NMR)-based metabolomics. The use of NMR for chemical structure determination and quantification of small molecules has a long history in successfully characterising the chemical composition within biological systems [25]. Furthermore, NMR-based metabolomics is useful in finding candidate active compounds through the correlation of activity patterns and the chemical profiles obtained from the NMR spectra [26].

The objective of this study was to identify bioactive antifungal compounds as potential biological control agents to control the plant pathogen *P. noxium* infecting heritage fig trees in Brisbane City, Australia. As part of the ongoing research on antifungal natural products derived from termite gut-associated *Streptomyces* isolates [9,27], this paper reports the identification of acetomycin (compound **1**) as a key component of the bioactive compounds in several termite gut-associated *Streptomyces* isolates with inhibitory activity towards plant fungal pathogen *P. noxium*.

## 2. Results and Discussion

### 2.1. Data Analysis of NMR Spectra from Fermentation Extracts of Streptomycetes

**Figure 1:**
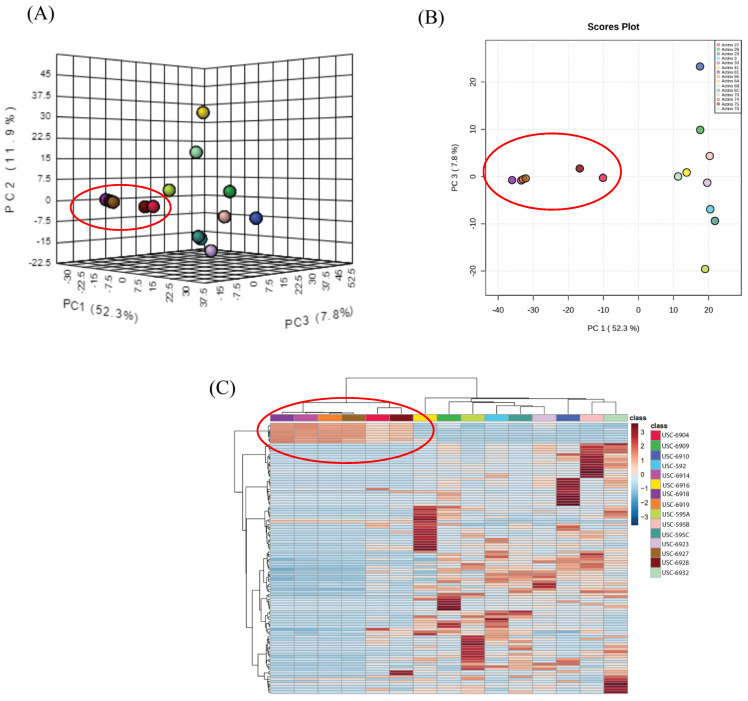
Data analysis of NMR spectra: (**A**) 3D and (**B**) 2-D score plot between selected PCs (explained variances shown in brackets) and (**C**) heatmap analysis of NMR peak intensities. The clustering results were measured using Euclidean distance and the clustering algorithm was measured using Ward distance.

The PCA models used in the present study efficiently captured the variations within the 1H-NMR spectra. This method of analyses enables reduction of the dimensionality of the data into inferred variables, therefore allowing for the identification of major trends and features. PC1 (52.3% variation), PC2 (11.9%), and PC3 (7.8%) demonstrated visible clustering from six of the fifteen streptomycetes fermentation extracts, including USC-6904, USC-6914, USC-6918, USC-6919, USC-6923, and USC-6928. Heatmap analysis clustered groups according to their metabolic profile and confirmed a strong correlation between these samples (Figure 1C).

**Figure 2:**
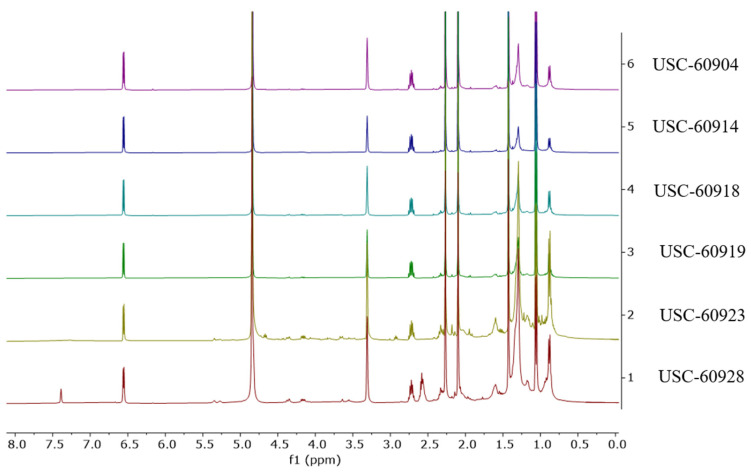
1H NMR spectra of extracts obtained from 6 *Streptomyces* isolates (USC-6904, USC-6914, USC-6918, USC-6919, USC-6923 and USC-6928).

Due to this observed similarity in the chemical profiles from the six streptomycetes fermentation extracts, the 1H NMR spectra were further investigated. The spectra confirmed the similarity of these extracts and revealed a high level of purity from the major compound (Figure 2). It can also be noted in Figure 1A,B, that isolates USC-6923 and USC-6928 have the biggest differentiation in their metabolites within the clustered six. This is also reflected in the NMR spectra as they contain the same major compounds, but the presence of other minor metabolites can be detected (Figure 2).

**Figure 3:**
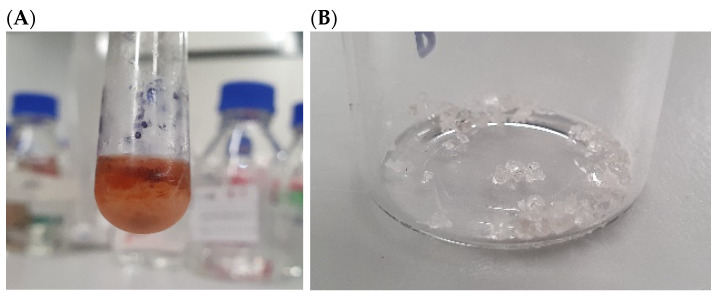
(**A**) Crystals recovered in the methanol solution of all six crude extracts pooled together and (**B**) compound **1** in a pure crystal form.

The cluster containing USC-6904, USC-6914, USC-6918, USC-6919, USC-6923, and USC-6928 from the NMR data in this study was comparable to the cluster obtained from the MS data analysis from our previous study using the same crude extracts. Additionally, these six isolates demonstrated the strongest activity out of the remaining fifteen and thus were investigated further [27]. However, 1H NMR spectra of these extracts were relatively simple with several signals of small molecules rather than signals of peptides or macrolides as determined by the MS-based molecular networking analysis [27]. The difference in a chemical composition conclusion between the NMR and MS methods led to further investigation of the bioactive compounds in these six active extracts. It was also noticed that these extracts had a formation of very small crystals (Figure 3A). As such, attempts at crystallisation were employed using various solvents, including hexane, dichloromethane, ethyl acetate, and methanol, in order to isolate these crystals from a pooling of all six crude extracts. Crystallisation using methanol was found to produce the largest crystal with the highest yield. The crystals were washed several times with cold hexane to gain sufficient purity (Figure 3B) for NMR and X-ray crystallography analyses.

### 2.2. Structure Elucidation for Compound **1**

**Figure 4:**
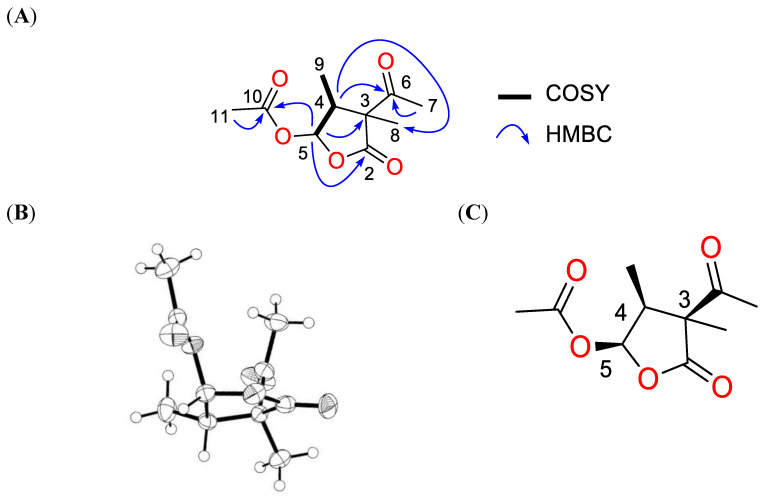
(**A**) Key COSY and HMBC correlations of compound **1**, (**B**) an ORTEP view of compound **1**, and (**C**) the absolute configuration of compound **1**.

Compound **1** was purified as colourless crystals and had a sodium adduct signal at (+) *m*/*z* 237.0734 in HR-ESI-MS corresponding to the molecular formula C_10_H_14_O_5_ with four degrees of unsaturation. The 1H NMR spectrum of compound **1** displayed three singlets (*δ_H_* H 2.27, 2.10, and 1.43), two doublets (*δ_H_* H 6.56 and 1.06), and one multiplet signal (*δ_H_* H 2.72) (Table 1). The 13C and edited HSQC experiments confirmed compound **1** had ten carbons, including one ketone carbonyl (*δ_C_* C 205.7), two ester carbonyls (*δ_C_* C 178.8 and 170.4), one acetal methine (*δ_C_* C 95.7), one quaternary carbon (*δ_C_* C 58.7), one methine (*δ_C_* C 46.2), and three methyl groups (*δ_C_* C 28.9, 20.4, and 9.7) (Table 1). A spin system was deduced from COSY correlations from the acetal methine proton at *δ_H_* H 6.56 (1H, d, *J* = 5.2 Hz, H-5) to the methine at *δ_H_* H 2.72 (1H, m, H-4) and *δ_H_* H 1.06 (3H, d, *J* = 7.6 Hz, H-9) (Figure 3A). The acetal proton H-5 showed long-range correlations to C-2 (*δ_C_* C 178.8), C-3 (*δ_C_* C 58.7), and C-10 (*δ_C_* C 170.4). Key HMBC correlations of H-4/C-6 and H-4/C-8 enabled the five-membered lactone ring to connect to the ketone C-6 (*δ_C_* C 205.7) and the methyl group C-8 (*δ_C_* C 20.8) at the quaternary carbon C-3. The downfield methyl groups C-7 (*δ_C_* C 28.9) and C-11 (*δ_C_* C 20.4) displayed HMBC correlations to C-6 and C-10, respectively, facilitating the establishment of the planar structure for compound **1** as a dihydrofuran-2(3H)-one derivative (Figure 4A). This compound has the trivial name acetomycin, which was first isolated from the cultures of Streptomyces ramulosus in 1958 [28]. To establish the absolute configuration of **1**, X-ray diffraction data were acquired using Cu K*α* radiation (Figure 4B), allowing for the unambiguous assignment of the absolute structure of **1** as (3*S*,4*S*,5*R*)-acetomycin, which was similar to the stereoisomer of acetomycin reported by Cano et al. [29] (Figure 4C).

Acetomycin has been reported to show weak antimicrobial activity against *Staphylococcus aureus*, *Candida albicans*, and *Bacillus subtilis* [30] and strong anticancer activity against HCT-8 human colon adenocarcinoma cells (IC_50_ of 1.5 µg/mL) and L1210 murine leukemia cells (IC_50_ of 2.2 µg/mL) [30]. Due to its rapid degradation caused by esterase-mediated hydrolysis, the compound becomes inactive in vivo [31]. As stated earlier, acetomycin was first isolated from *S. ramulosus*, whereas this study can now report its isolation for the first time from isolates USC-6904 and USC-6914 (close relatives of *S. catenulae*), USC-6918 (close relative of *S. luozhongensis*), USC-6919 (close relative of *S. thermocarboxydus*), USC-6923 (close relative of *Streptomyces* sp. CX3), and USC-6928 (close relative of *S. diastaticus*) according to the 16S oligonucleotide analysis and phylogeny of these isolates reported from our previous study [27].

### 2.3. Assessing the Activity of Acetomycin against P. noxium Using the Disk Diffusion Method

**Figure 5:**
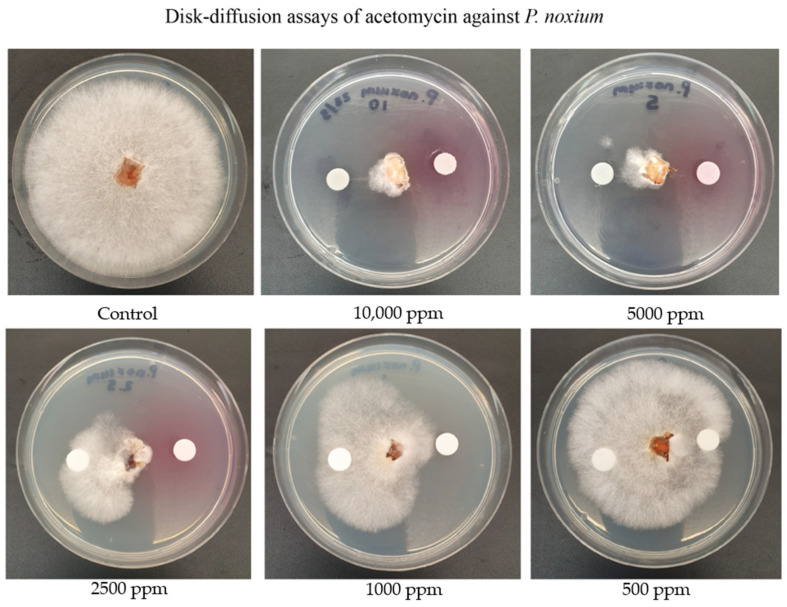
Disk diffusion assay assessing the antifungal activity of acetomycin against *P. noixum* at varying concentrations.

The activity of the recovered acetomycin crystals against the *P. noxium* was evaluated using the Kirby–Bauer disk diffusion method (Figure 5). Strong activity was observed from at 10,000 ppm (10 µg/mL), 5000 ppm (5 µg/mL), 2500 ppm (2.5 µg/mL), and 1000 ppm (1 µg/mL), although around 500 ppm activity was very modest. Acetomycin was previously tested for antifungal activity against the plant pathogens *Piricularia oryzae* (which causes rice blast) and *Botrytis cinerea* (which causes bunch rot in grapes) [30]. Antifungal activity towards *B. cinerea* was only present at 10,000 ppm; however, for *P. oryzae*, activity was present at 100 ppm [30]. No previous research has been found assessing the antifungal activity of this antibiotic against *P. noxium* nor investigating the potential of this antibiotic as a biological control agent.

### 2.4. Assessing Acetomycin for Antifungal Activity against Other Fungal Crop Pathogens

The acetomycin was further tested for antifungal activity against other important fungal crop pathogens, including *Botrytis cinerea*, *Aspergillus niger*, and *Alternaria alternata*, and their MIC values were determined (Table 2). The addition of these crop pathogens was to demonstrate this compounds effectivity and wide-spectrum activity against other fungal pathogens that similarly cause much destruction within the agricultural industry. Activity was defined as per the growth patterns in Figure 5 and following the same methods. *B. cinerea* causes bunch rot in grapes and has also infested chickpeas within Australia [32]. *A. niger* is responsible for the post-harvest decay of fresh fruit, including grapes, and has caused issues for vineyards within New South Wales, Australia [33]. *A. alternata* causes black spot in many fruits and vegetables and has infected cotton seedlings also grown in New South Wales in Australia [34,35,36]. As mentioned above, Acetomycin has been previously assessed against *B. cinerea* [30]; however, activity was only observed at 10,000 ppm. Within this study, the inhibition of acetomycin against *B. cinerea* was observed at 10,000 ppm, as well as at 5000 ppm and 2500 ppm. No previous research has assessed acetomycin for antifungal activity against *Aspergillus niger* and *Alternaria alternata*. *A. niger* demonstrated antifungal activity with an MIC of 1000 ppm, while *A. alternata* displayed an MIC of 500 ppm. Therefore, acetomycin can demonstrate antifungal activity against a wide range of fungal crop pathogens and also has potential as a strong biological control agent against the *A. alternata* fungal pathogen.

### 2.5. In Vitro Evaluation of the Inhibitory Potential of Acetomycin on P. noxium Using a Woodblock Test

**Figure 6:**
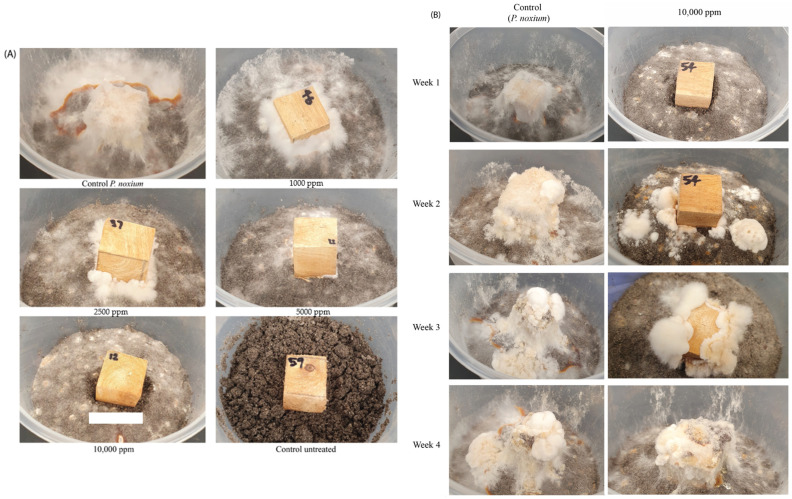
(**A**) Growth of *P. noxium* on woodblocks after 1 week of acetomycin treatment at various concentrations. (**B**) Comparison of the *P. noxium* control woodblock and the acetomycin-treated woodblock at 10,000 ppm over 4 weeks.

**Figure 7:**
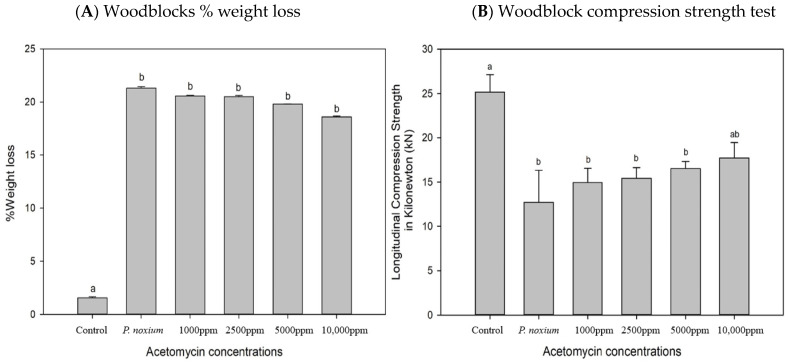
The (**A**) %weight loss and (**B**) the compression strength test of the woodblocks. Error bars represent standard error of the mean, and statistically significant (*p* < 0.05) differences are noted by different letters (a and b above the bars) according to Tukey’s test.

To assess the in vitro antifungal activity of acetomycin against *P. noxium*, the woodblock test was employed [11]. Figure 6A compares the antifungal activity of acetomycin against *P. noxium* at selected active concentrations, whilst Figure 6B compares the activity of the 10,000 ppm concentration to the *P. noxium* control over a four-week period. A clear difference could be observed between the *P. noxium* control woodblocks and the treated ones. At 10,000 ppm, a clearance zone in the soil and around the woodblocks could be remarkably observed (Figure 6A,B). Additionally, within the first three weeks, a visible difference could be noted when comparing the woodblocks containing 10,000 ppm to the control; however, by week four, the fungus had fully covered the woodblocks, indicating loss of activity (Figure 6B). At week six, due to full fungal resurgence, the experiment was terminated, and the %weight loss and the strength test were conducted on the woodblocks. The %weight loss values for the treated woodblocks were not significantly different from each other or from the *P. noxium* control, with the only significant difference existing between the untreated control woodblocks (F (5,30) = 71.201) *p* ≤ 0.001). However, a slight decrease in weight loss can be observed as the concentration of acetomycin increases. *P. noxium* lost an average of 21.6% (1.57 g), the 1000 ppm sample lost an average of 20.6% (1.54 g), the 2500 ppm sample lost an average of 20.5% (1.52 g), the 5000 ppm sample lost an average of 19.8% (1.51 g), and the 10,000 ppm sample lost an average of 18.6% (1.36 g) (Figure 7A). However, the results of the compression strength test presented with significant differences between the acetomycin-treated woodblocks and the *P. noxium* control (F (5,30) = 20.186) *p* = 0.001). The *P. noxium* control required an average force (kN) of 10.88, the 1000 ppm sample required 16.34, the 2500 ppm sample required 17.55, the 5000 ppm sample required 19.01, and the 10,000 ppm sample required 20.09 (Figure 7B). By week four, the activity of the acetomycin was lost; however, the results from the compression strength test reveal a significant amount of protection for the woodblocks against the *P. noxium* pathogen.

The eventual loss of activity from acetomycin may be explained by potential hydrolysis of the ester functional group by the enzyme esterase. Previous studies have investigated this antibiotic for its demonstrated anti-tumour biological activity and found it became inactivated in an in vivo assay due to the production of this enzyme [31,37]. Additionally, *P. noxium* has been reported to produce esterase enzymes [38,39] as a mechanism to break down xylan [40,41] from the wood of the tree host. Attempts have been made to design and synthesise esterase-resistant analogues of acetomycin. In 1999, Uenishi et al. synthesised analogues with benzoyloxy and pivaloyloxy groups instead of the acetoxy group at the 5-position of the γ-butyrolactone ring; however, activity was still only observed in an in vitro assay [37]. Further attempts were made in 2004 with acetomycin bislactone analogues, and they were able to successfully report these derivatives to be completely resistant to esterase; however, their cytotoxicity was reduced compared to the parent antibiotic [42]. Further investigation is required to better understand the structure/activity relationship between the chemical and the *P. noxium* fungus and whether or not the pathogen is indeed producing esterase to break down this compound. This could be achieved by repeating the woodblock test but with esterase-resistant analogues to assess for increased stability of the compound in the presence of the fungal pathogen.

Acetomycin represents a unique antibiotic with a remarkable density within the three adjacent chiral centers at the lactone ring system and a diversity of antimicrobial activity, including antibacterial, antitumour, and antifungal activity. Within this study, strong antifungal activity against *P. noxium* was observed for this antibiotic; however, it was observed that during the woodblock assay, antifungal activity ceased with time. This means that, in terms of potential application as a fungicidal product, it would require reapplication. Although this can be seen as a limitation, its degradability in nature is what makes natural product fungicides the first option to be used by agriculturalists and plant biologists for combating fungal pathogenesis [43], particularly in comparison to persistent toxic chemicals in our environment and within agricultural crops. A recent review paper listed “readily degradable” as a desirable feature of new fungicides and that they should leave minimal residue in the agricultural produce [44]. In fact, recent years have seen the direct application or structure modification of natural products as effective ways to discover and synthesise new pesticides [45]. They are able to be used as products in their own right or often can present as new chemical skeletons that can be modified by synthetic chemists or as indicators of new and effective biochemical modes of action [46]. An example of this is the natural product fungicide Blasticidin-S, which was isolated from *Streptomyces griseochromogenes* in 1955 by Fukunaga et al. It is a contact fungicide used to control rice blast caused by *P. oryzae* and is relatively non-hazardous to non-target organisms and has no deleterious effects on the environment [46,47]. Therefore, acetomycin may similarly have the potential to act as a natural product fungicide against *P. noxium* with the ability to degrade without harmful residues remaining.

Not much is known about the mechanisms of action from this compound. One study investigated the structure–activity relationship of acetomycin and conjectured that the lactone carbonyl plays an important role in the observed biological activity, as well as the ester functionality at C-5 [48]. This was determined through synthesis of multiple analogues and identification of changes to activity in tumour cells [48]. Unfortunately, there are a lack of data that investigate the mode of action of acetomycin and a similar lack of any molecular data on the effects it has against pathogenic plant organisms. Future directions of this study should include investigations into the molecular bases of the mode of action and the physiological processes it has on the effected fungi of this study.

## 3. Materials and Methods

### 3.1. Cultures and Culture Media

The 15 selected actinomycete isolates used in this study were previously screened for activity in our previous study [27] and were identified as the most active out of 37 actinomycetes. The actinomycete isolates were isolated from the guts of termites, *Coptotermes lacteus* (Froggatt) species located in the Sunshine Coast Region of Queensland, Australia [36,37], and cryogenically preserved in the University of the Sunshine Coast Microbial Library. The 16S analyses of these isolates were conducted in our previous study [27]. The actinomycetes were grown onto oatmeal agar [49], and the *P. noxium* strain was previously isolated from infected heritage fig trees within Brisbane, Queensland, and grown on potato dextrose agar (PDA) (OXOID, Thebarton, Australia).

### 3.2. Fermentation and Extraction of Metabolites Produced by Streptomyces Isolates

Fermentation of the *Streptomyces* isolates was conducted using solid-state fermentation. The isolates were inoculated onto oatmeal agar plates and incubated for 7 days at 28 °C. The plates were then inoculated with a fungal plug from the *P. noxium* and grown for three more days using the same conditions. These plates were then twice extracted using ethyl acetate (ChemSupply, Sydney, Australia) (EtOAc) by cutting up the agar and flooding it with the solvent for 24 h. The solvent was filtered and evaporated under a vacuum to obtain the crude extract for further testing [27].

### 3.3. Nuclear Magnetic Resonance Analysis

NMR profiling of the crude extracts was conducted by solubilising the crude extracts in methanol-d4 at a concentration of 10 mg/mL. NMR spectra were acquired on a Bruker Ascend 400 spectrometer (Bruker, Karlsruhe, Germany) equipped with a 5 mm room temperature probe operating at 400 MHz for 1H spectra and 100 MHz for 13C spectra. The ^1^H and ^13^C spectra were referenced to the residual deuterated solvent peaks at *δ_H_* 3.31 and *δ_C_* 49.0 (methanol-d4). HRESIMS data were acquired on a Sciex X500R Q-TOF mass spectrometer (Sciex, Framingham, MA, USA). HPLC purifications were performed on a preparative Agilent 1200 system (Agilent Technologies, Palo Alto, CA, USA) equipped with a diode array detector and processed by ChemStation software (C.01.07). All solvents used for extraction and chromatography were HPLC grade and the H_2_O used was Mili-Q water.

(3*S*,4*S*,5*R*)-acetomycin (**1**): colourless crystals; 1H and 13C NMR data, Table 1; (+) HR-ESI-MS *m*/*z* 237.0734 [M + Na]^+^ (calcd for C_10_H_14_O_5_Na^+^, 237.0733, Δ 0.4 ppm).

### 3.4. Crystallographic Data

Acetomycin: C_10_H_14_O_5_; MW 214.21; T 190 K; orthorhombic space group; P212121: unit cell dimensions: a = 7.1535(4) Å, b = 10.5157(6) Å, c = 14.0844(7) Å, V 1059.49(10) Å3, α = 90°, β= 90°, γ = 90°, Z = 8, ρcalcd = 1.343 mg/m^3^, µ = 0.917 mm^−1^, F(000) = 456; crystal dimensions: 0.20 × 0.08 × 0.05 mm^3^. The 4049 measured data points yielded 1634 independent reflections (R(int) = 0.0495). The final refinement resulted in R1 = 0.0440 and wR2 = 0.1017 (all data), goodness of fit 1.071.

### 3.5. Structure Determination

X-ray diffraction analysis was carried out on an Oxford Diffraction Gemini S Ultra CCD diffractometer using Cu Kα radiation (λ = 1.54180 Å), and data were collected within the 5.249° < θ < 61.589° scan range. The crystal was preserved at a temperature of 190 K using an Oxford Cryosystems Desktop Cooler (Oxford, UK). Data reduction and semi-empirical absorption corrections were conducted with the CrysAlisPro software (v42) package (Rigaku-Oxford Diffraction, Yarnton, Oxfordshire, UK). The structure was solved by direct methods and refined by full-matrix least-squares on F2 with SHELX [50] within the WinGX graphical user interface [51]. ORTEP3 was used to produce the thermal ellipsoid plots [52]. The absolute structure was established from a statistical analysis of anomalous dispersion effects of 10,150 Bijvoet pairs [53] with PLATON [54], leading to a probability (P2) of the finding being the correct enantiomer of 1.00.

### 3.6. Assessing Activity from Acetomycin against P. noxium and Other Fungal Crop Pathogens

Purified acetomycin was assessed for antifungal activity against *P. noxium* and other fungal crop pathogens, including *B. cinerea*, *A. niger*, and *A. alternata*, using the Kirby–Bauer disk diffusion technique [55]. The acetomycin crystals were solubilised in ethyl acetate (EtOAc) at varying concentrations in order to obtain the minimum inhibitory concentration (MIC). The concentrations are as follows: 10,000 ppm, 5000 ppm, 2500 ppm, 1000 ppm, 500 ppm, and 100 ppm. The dissolved acetomycin was then seeded onto a sterile paper disk and placed onto a PDA plate beside a fungal plug of the selected fungal pathogens. A blank sterile paper disk containing only EtOAc was placed on the opposite side of the fungal plug as a control. This was completed for all concentrations and placed into a 28 °C incubator for 3 days. Signs of growth inhibition from the fungal mycelium were observed and compared to a control plate containing only a fungal plug and two blank sterile paper disks as well as the blank disk on the same plate.

### 3.7. Assessing Acetomycin as a Potential Biocontrol against P. noxium Using Woodblocks

To assess the biocontrol potential and stability of acetomycin, a woodblock study was conducted. Woodblocks (2 cm × 2 cm) were freshly cut from a healthy Moreton Bay fig tree (*Ficus macrophylla*) and dried in a 60 °C oven until a constant dry weight was obtained. The dry weight of each woodblock was recorded before being autoclaved twice at 121 °C for 40 min on a dry cycle to prevent the accumulation of moisture. Topsoil was also twice sterilised under the same conditions and 200 g of the soil was added into sterile polypropylene containers with air filter-embedded lids. The soil moisture level was maintained at 65% water holding capacity through the addition of sterilised distilled water and mixing. Millet seeds inoculated with the *P. noxium* were then added to the soil (2%) and again mixed. The sterilised woodblocks were then placed into the centre of the containers on top of the soil. Blocks were then treated with the acetomycin at varying concentrations. Acetomycin was dissolved into 1 mL of EtOAc at concentrations of 10,000 ppm, 5000 ppm, 2500 ppm, and 1000 ppm (concentrations were selected based on the results of the previous disk diffusion assay). The dissolved compound was pipetted onto each face of the block (150 µL each side and 250 µL on the bottom face where it is in direct contact with the pathogen). There were six replicates for each concentration, and controls were also established by using six blocks without the acetomycin treatment and six blocks with no acetomycin treatment nor *P. noxium*-infected millet seeds. These containers were then placed in the 28 °C incubator for 6 weeks. The blocks were then cleaned and sterilised and placed back in the 60 °C oven to obtain the dry weight before comparison with the original dry weight, allowing for the percentage of weight loss to be calculated. The blocks also underwent a compression strength test using a metal jig of an Instron testing machine (Shimadzu Autograph AG-X plus (Shimadzu, Tokyo, Japan), 100 kN), where the longitudinal axis of the wood was set parallel to the loading direction. The wood samples were compressed at a crosshead speed of 0.3 mm/min. The compressive strength test entailed compression to 5% of the original dimension of the woodblock, with the result subsequently recorded. The load required for this compression was used as a measure of residual strength. All the woodblocks had the same surface area exposed to the loading and were compressed to the same percentage of their length. LCS values were recorded as newtons (N) [11].

### 3.8. Statistical Analysis

The web-based tool MetaboAnalyst 5.0 (https://www.metaboanalyst.ca/, accessed on 13 June 2023) was used for processing, analysis, and interpretation of the 1H NMR spectral data. The datasets were subjected to Multivariate Principal Component Analysis (PCA) and heatmap analysis to estimate the differences in chemical profiles amongst the fermentation extracts. SPSS software version 29.0 (IBM Corp., Armonk, NY, USA) was used to analyse the results from the woodblock study for both %weight loss and the compression strength test. Prior to the analysis, the normality of data distribution was assessed using the Shapiro–Wilk test and the homogeneity of variances was determined using Levene’s test. One-way ANOVA was conducted to test significant differences between the means. When homogeneity of variance was proven, post hoc pairwise comparisons were performed using Tukey’s test. However, when homogeneity of variance was not proven, the Games–Howell test was used. All statistical analyses were performed at a significance level of *p* ≤ 0.05.

## 4. Conclusions

Using NMR-based metabolomic methods, the chemical profiles of 15 termite gut-associated streptomycetes were investigated. Chemical structure similarities within six of the isolates led to the isolation of the known compound acetomycin. This study describes for the first time the antifungal activity of acetomycin against *P. noxium*, *A. niger*, and *A. alternata.* The woodblock test confirmed the biological control potential of acetomycin against *P. noxium* as strong antifungal activity was observed for up to 3 weeks. This compound’s ability to degrade with time meets the requirements of biological control agents as opposed to toxic and persistent chemical control fungicides. Future research directions could investigate the mode of action of acetomycin on *P. noxium* and also the orchestration of field trials to assess its efficacy for field application.

## Figures and Tables

**Table 1:** NMR spectroscopic data (^1^H 400 MHz, ^13^C 100 MHz) in methanol-d4 for **1**.

Position	*δ_C_*	Multiplet	*δ_H_* (*J* in Hz)	HMBC
2	178.8	C		
3	58.7	C		
4	46.2	CH	2.72, m	3, 5, 6, 8, 9
5	95.7	CH	6.56, d (*J* = 5.2)	2, 3, 4, 10
6	205.7	C		
7	28.9	CH_3_	2.27, s	3, 6
8	20.8	CH_3_	1.43, s	2, 3, 4, 6
9	9.7	CH_3_	1.06, d (*J* = 7.6)	3, 4, 5
10	170.4	C		
11	20.4	CH_3_	2.10, s	10

**Table 2:** Antifungal activity of acetomycin against other important fungal crop pathogens.

Fungal Crop Pathogen	10,000 ppm	5000 ppm	2500 ppm	1000 ppm	500 ppm
*Botrytis cinerea*	+++	++	+	-	-
*Aspergillus niger*	++	+	+	+	-
*Alternaria alternata*	+++	+++	++	+	+

+++ = strong antifungal activity, ++ = antifungal activity, + = minimal activity, - = no activity.

## Data Availability

Data are contained within the article.
